# A Warning in Regard to the Reduction of Dislocation of the Humerus

**Published:** 1899-03-25

**Authors:** 


### A WARNING IN REGARD TO THE REDUCTION OF DISLOCATION OF THE HUMERUS.

At a recent meeting of the Liverpool Medical Institution Dr. Nathan Raw exhibited a patient on whom he had operated for traumatic aneurism of the axilla. This he had treated by free incision, with ligature of the axillary vein and subscapular artery, after placing a temporary ligature on the first part of the axillary artery and vein. The case is interesting, not only on account of the successful issue to what Dr. Mitchell Banks described as a "dangerous, difficult, and trying case," but as conveying a warning about the use of the foot in the axilla as a means of reducing a dislocation of the humerus, for this proceeding was the cause of the mischief in the case in question.

